# *In vitro* activity of 1H-phenalen-1-one derivatives against *Leishmania* spp. and evidence of programmed cell death

**DOI:** 10.1186/s13071-019-3854-4

**Published:** 2019-12-23

**Authors:** Atteneri López-Arencibia, María Reyes-Batlle, Mónica B. Freijo, Ines Sifaoui, Carlos J. Bethencourt-Estrella, Aitor Rizo-Liendo, Olfa Chiboub, Grant McNaughton-Smith, Jacob Lorenzo-Morales, Teresa Abad-Grillo, José E. Piñero

**Affiliations:** 1 Instituto Universitario de Enfermedades Tropicales y Salud Pública de Canarias, Universidad de La Laguna, Avda. Astrofísico Fco. Sánchez, S/N, 38203 La Laguna, Tenerife, Islas Canarias Spain; 2 Departamento de Obstetricia y Ginecología, Pediatría, Medicina Preventiva y Salud Pública, Toxicología, Medicina Legal y Forense y Parasitología, Universidad de La Laguna, La Laguna, Tenerife Spain; 3 Instituto Universitario de Bio-Orgánica ‘Antonio González’, Departamento de Química Orgánica, Universidad de La Laguna, Avda. Fco. Sánchez 2, 38206 La Laguna, Tenerife, Islas Canarias Spain; 4 Laboratoire Matériaux-Molécules et Applications, La Marsa, University of Carthage, Carthage, Tunisia; 5 Centro Atlántico del Medicamento S.A (CEAMED S.A.), PCTT, La Laguna, Tenerife, Islas Canarias Spain

**Keywords:** *Leishmania donovani*, *Leishmania amazonensis*, Phenalenones, Chemotherapy, Apoptosis-like

## Abstract

**Background:**

The *in vitro* activity against *Leishmania* spp. of a novel group of compounds, phenalenone derivatives, is described in this study. Previous studies have shown that some phenalenones present leishmanicidal activity, and induce a decrease in the mitochondrial membrane potential in *L. amazonensis* parasites, so in order to elucidate the evidence of programmed cell death occurring inside the promastigote stage, different assays were performed in two different species of *Leishmania*.

**Methods:**

We focused on the determination of the programmed cell death evidence by detecting the characteristic features of the apoptosis-like process, such as phosphatidylserine exposure, mitochondrial membrane potential, and chromatin condensation among others.

**Results:**

The results showed that four molecules activated the apoptosis-like process in the parasite. All the signals observed were indicative of the death process that the parasites were undergoing.

**Conclusions:**

The present results highlight the potential use of phenalenone derivatives against *Leishmania* species and further studies should be undertaken to establish them as novel leishmanicidal therapeutic agents.
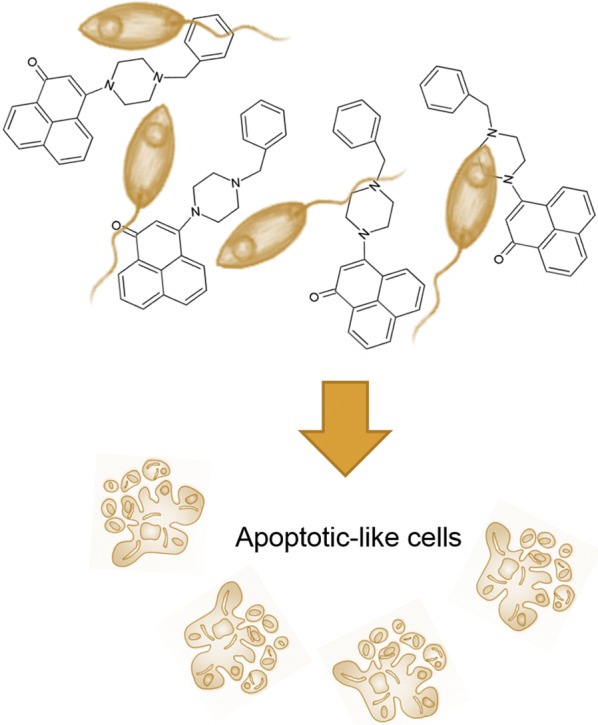

## Background

Leishmaniasis is a parasitic disease caused by the obligate intracellular parasites of the genus *Leishmania* which are transmitted to human by the bite of a female sand fly [1]. According to the World Health Organization (WHO), leishmaniasis is one of the seven most important tropical diseases and it represents a serious world health problem that presents a broad spectrum of clinical manifestations with a potentially fatal outcome [2, 3]. It is found in all continents except for Oceania and Antarctica [2, 4] and is endemic in circumscribed geographical areas in north-eastern Africa, southern Europe, the Middle East, south-eastern Mexico, and Central and South America.

Pentavalent antimony-based medicines for leishmaniasis have been in clinical practice from 1947 [5] but they cause side effects. The efficacy of pentavalents has decreased and the emergence of resistance limits their usage [6, 7]. The first line drugs in leishmaniasis including meglumine antimoniate (glucantime) and sodium stibogluconate (pentostam) are not effective orally and require prolonged injections. The second line drugs such as amphotericin B and pentamidine are very toxic [6]. In the absence of an effective vaccine, there is an urgent need for new and more effective drugs to replace or supplement those in current use. Natural and synthetic 1H-phenalen-1-one (PH) containing compounds exhibit a diverse range of biological activities, antifungal [8–12], antiprotozoal [10, 13–15], bactericidal [16] and cytotoxic activity against human cancer cells [17]. The active 1H-phenalen-1-one analogs were evaluated against *Leishmania* spp. in order to determine the programmed cell death evidence [18].

Induction of programmed cell death (PCD) in parasites is a novel possibility for drug development that has been explored in great detail in several unicellular parasitic protozoans [19]. PCD in unicellular organisms has been reported so far in yeasts, *Dictyostelium discoideum*, *Peridinium gatuense*, *Euglena gracilis*, *Tetrahymena thermophila*, and parasites like *Trypanosoma* spp., *Leishmania* spp., *Plasmodium* spp., *Blastocystis hominis* and *Entamoeba histolytica* [19–23]. In these organisms, these apoptotic processes occur as a phenomenon that presumably benefits the rest of the population in some way. The fact that there is evidence that apoptotic processes exist in protozoan parasites has provided new strategies for the development of tools in the study of these diseases, comparing these processes to those in humans [19, 22–24].

Several typical markers of mammalian apoptosis have been found in *Leishmania* spp., suggesting the existence of an apoptosis-like death in these organisms. The markers include: cell shrinkage; nuclear chromatin condensation; DNA fragmentation; membrane blebbing; mitochondrial transmembrane potential loss; and phosphatidylserine exposure [25]. In addition, *Leishmania* cell death appears very peculiar, different stimuli can induce a form of cell death with the same phenotypic features as mammalian apoptosis that we can call *Leishmania* apoptosis [26].

In the present study, the *in vitro* effects at the cellular death level of various 1H-phenalen-1-one derivatives were evaluated against two species of *Leishmania*. The results highlight a potential use of these active compounds for the treatment of leishmaniasis in the near future.

## Methods

### Chemicals

**Fig. 1:**
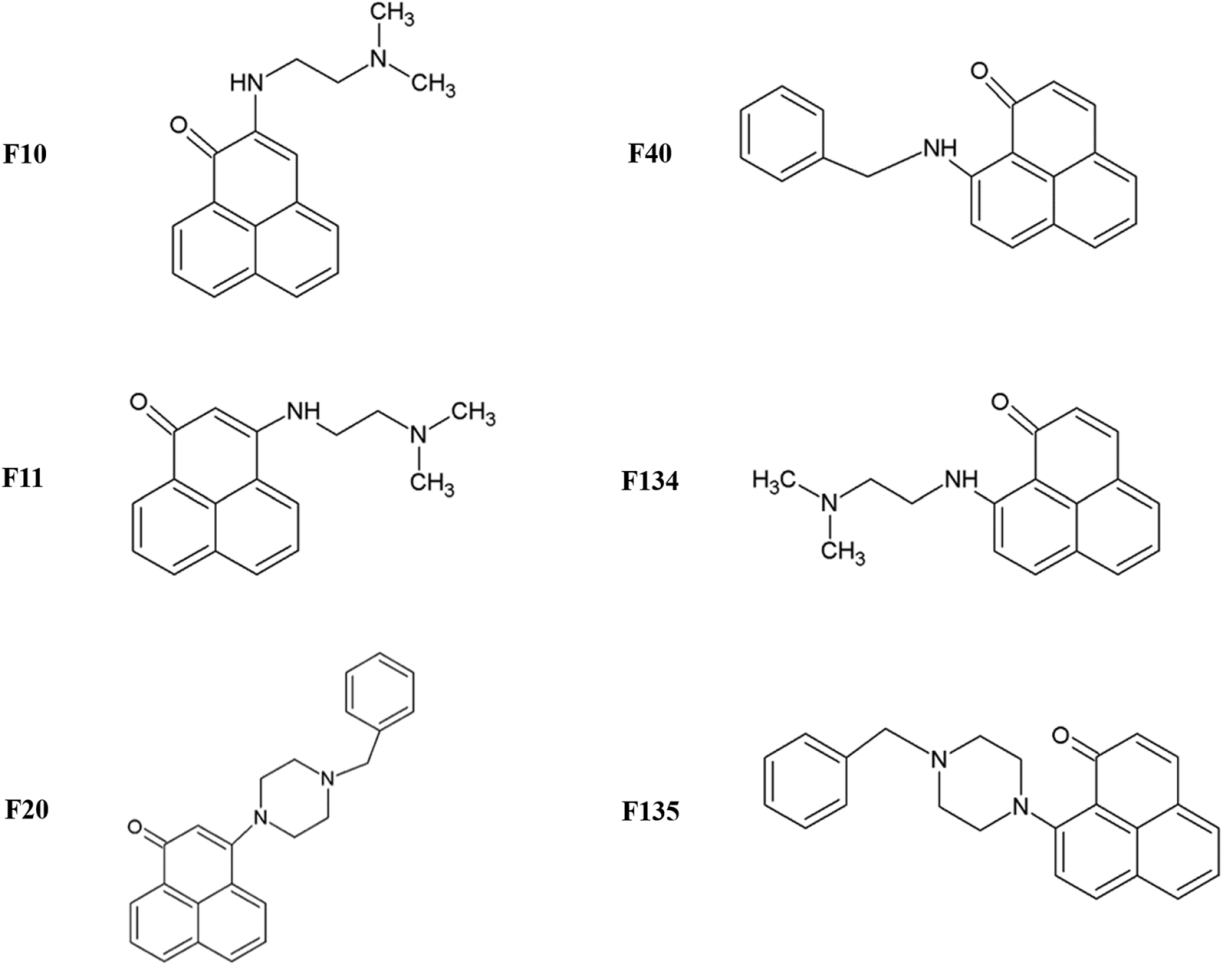
Structure of the evaluated 1H-phenalen-1-one derivatives

All compounds tested, shown in Fig. 1, were synthesized as described by Blanco-Freijo et al. [18]. Stock solutions of these drugs were prepared in dimethyl sulfoxide (DMSO) (Merck, Darmstadt, Germany), protected from the light, at − 20 °C until required.

### Parasites

Experiments were carried out with *Leishmania amazonensis* (MHOM/BR/77/LTB0016) and *Leishmania donovani* (MHOM/IN/90/GE1F8R) strains. Promastigotes of both strains were cultured in Schneider’s medium (Sigma-Aldrich, Darmstadt, Germany) supplemented with 10% fetal bovine serum at 26 °C and were grown to the log phase. For some of the assays, the parasites were also cultured in RPMI 1640 medium (Gibco, Massachusetts, USA), with or without phenol red.

### Analysis of ATP levels

ATP levels were measured using a Cell Titer-Glo*®* Luminescent Cell Viability Assay (Promega, Wisconsin, USA), which generates a proportional signal in relation to the amount of ATP. Promastigotes were incubated with the IC_90_ of the different 1H-phenalen-1-one derivatives for 24 h. Aliquots were taken and mixed with the kit reagent into white plates following the manufacturerʼs instructions for posterior measurement of the luminescence on a PerkinElmer spectrophotometer (PerkinElmer, Massachusetts, USA).

### Analysis of mitochondrial membrane potential

The collapse of an electrochemical gradient across the mitochondrial membrane during apoptosis was measured using a JC-1 Mitochondrial Membrane Potential Assay Kit (Cayman Chemical, Michigan, USA). After being treated with IC_90_ concentrations of the tested molecules for 24 h, the promastigotes were centrifuged (1500× *rpm* for 10 min) and resuspended in JC-1 buffer. Thereafter, 100 μl of each treated culture was added to a black 96-well plate than 10 μl of JC-1 was added and incubated at 26 °C for 30 min. Mean green and red fluorescence intensity was read using an Enspire microplate reader (PerkinElmer) for 30 min. Data are expressed as the ratio 595/535 (J-aggregates:J-monomers) as an indicator of cell health.

### Plasma membrane permeability

Sytox green nucleic acid stain (Life Technologies, Massachusetts, USA) is a high-affinity nucleic acid stain (absorption and emission maxima at 504 and 523 nm, respectively) that renders cells with compromised plasma membranes bright fluorescent green. The experiment was carried following the manufacturer’s recommendations. Briefly, 10^7^ cells/ml were seeded in a black-wall 96-well microtiter plate (Thermo Fisher Scientific, Massachusetts, USA) with the previously calculated IC_90_s of each active principle. A negative control (without treatment) and a positive control (with 2.5% of Triton X-100 (Sigma-Aldrich)) were added in order to obtain fully permeabilized cells. Measurements were performed by using an EnSpire microplate reader (PerkinElmer) every 15 min for 6 h, after which parasite samples were removed for fluorescence microscopy analysis.

### Phosphatidylserine externalization

An Annexin V/Propidium Iodide (PI) double-staining assay (Life Technologies) was performed using a TALI apoptosis kit, Annexin V-Alexa Fluor 488 and propidium iodide according to the manufacturer’s instructions. Briefly, after being treated with the IC_90_ for 24 h, promastigotes were washed twice with the annexin binding buffer (ABB), and incubated with 5 μl of annexin V for 20 min. After that, cells were centrifuged at 1500× *rpm* for 10 min and resuspended in ABB containing 1 μl of PI and incubated for 3 min at room temperature. Finally, a 25 μl volume of the stained cells were loaded into a TALI^TM^ cellular analysis slide and for analysis in a TALI™ image-based cytometer. Data were collected using TALI™ data acquisition and analysis software (Life Technologies). Data are expressed in percentages of the total population analyzed on each sample, divided into three groups: apoptotic cells (green fluorescence); dead cells (red or red and green fluorescence); and live cells (no fluorescence).

### DNA condensation

For the detection of condensed chromatin the Vybrant™ Apoptosis Assay Kit #5, Hoechst 33342/Propidium Iodide (Life Technologies) was utilized. Parasites were treated with the IC_90_ of the PHs for 24 h, then harvested and centrifuged at 1500× *rpm* for 10 min. The cell pellet was resuspended in RPMI, added to a black 96-well plate and incubated with the Hoechst 33342 and the propidium iodide. Then the plate was protected from light for 20 min at 4 °C. The EVOS FL Cell Imaging System (Thermo Fisher Scientific) was used to observe the cells, using the DAPI (Hoechst) and RFP (PI) Light Cubes.

### Oxidative stress

CellRox Deep Red Oxidative Stress Reagent (Thermo Fisher Scientific) is a probe designed to measure reactive oxygen species (ROS) in live cells, exhibiting strong fluorogenic signal under oxidative state. Following the manufacturer’s protocol, parasites were treated with the IC_90_ for 24 h, then washed, and incubated with 5 μM of CellRox Reagent for 30 min at 26 °C. After that, cells were centrifuged at 1500× *rpm* for 10 min and resuspended in 50 µl of buffer’s kit. Parasites treated with H_2_O_2_ at 600 μM for 30 min were used as a positive control [27]. A fluorescence microscope (Leica TCS; Leica Microsystems, Wetzlar, Germany) was used to observe the cells, using an excitation wavelength of 640 nm and emission wavelengths of 665 nm, the signal for Deep Red which should appear localized in the cytoplasm.

### Statistical analysis

Data are presented as the mean ± standard error (SE). All determinations were performed in triplicate and the data shown are representative results from at least three independent experiments. Statistical differences between means were tested using a one-way analysis of variance (ANOVA; three or more samples), checking for both normality and homoscedasticity, with a *post-hoc* pairwise comparisons of means carried out using Tukeyʼs test, using the SigmaPlot 12.0 software. A significance level of *P* < 0.05 was used.

## Results

### Mitochondrial effects

**Fig. 2:**
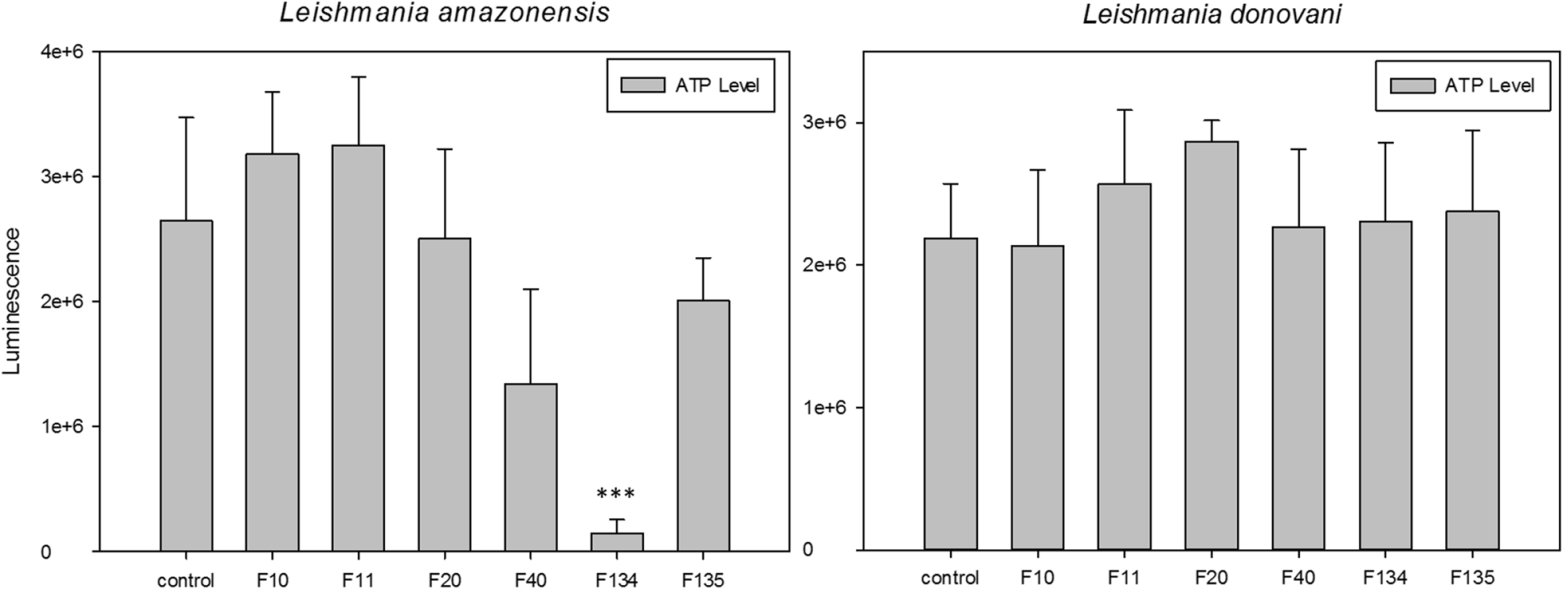
ATP levels in relative luminescence units (RLU) of *L. amazonensis* and *L. donovani* after 24 h of incubation with the IC_90_ of the molecules. Error bars represent the standard deviations (SD). Each data point indicates the mean of the results of three measurements. ***P < 0.001

We observed that incubation with phenalenone F134 induced a highly significant decrease of the ATP level of *L. amazonensis* promastigotes (*F*_(6, 20)_ = 10.504, *P* = 0.002). The remaining compounds did not show any alteration in the ATP levels of the promastigotes after 24 h of incubation with the IC_90_ of the molecules (Fig. 2).

**Fig. 3:**
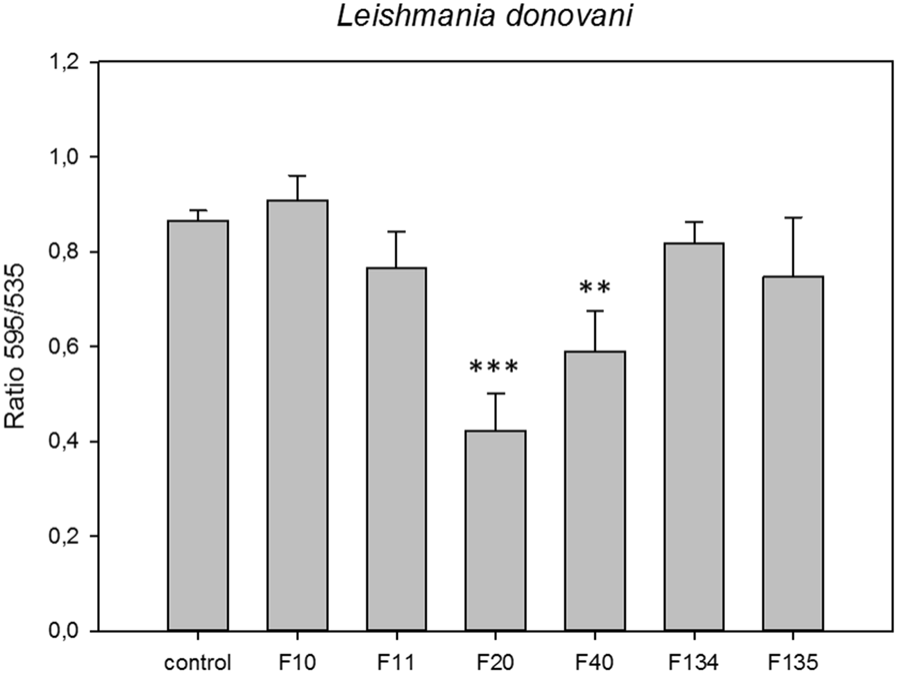
Changes in the mitochondrial membrane potential (ΔΨm) of *L. donovani* after 24 h of incubation with the IC_90_ of the phenalenones. Error bars represent the standard deviations (SD). Each data point indicates the mean of the results of three measurements. **P < 0.01, ***P < 0.001

In the present study we also examined the mitochondrial membrane potential alterations in *L. donovani*, and the results showed that the membrane potential also decreased when incubated with the IC_90_ of the phenalenones F20 and F40 (Fig. 3) (*F*_(6, 20)_ = 15.083, *P* < 0.001 and *F*_(6, 20)_ = 15.083, *P* = 0.008, respectively).

### Membrane alterations

**Fig. 4:**
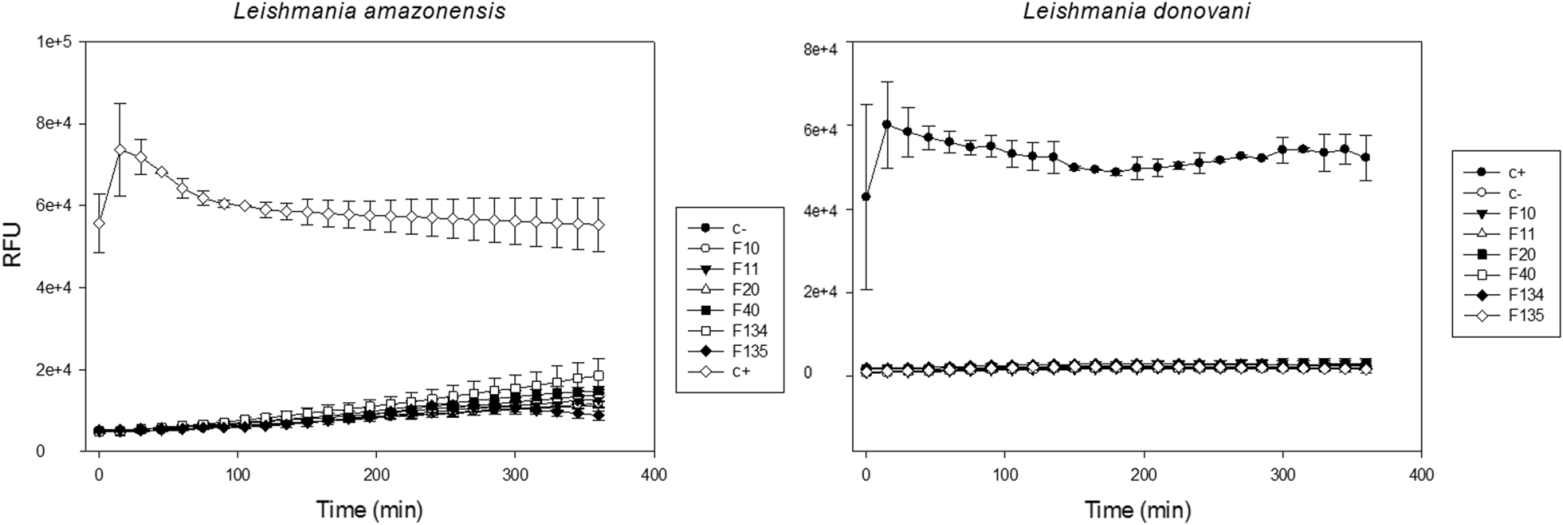
Plasma membrane permeability assay results. RFU are the relative fluorescent units. A strong increase in the RFU will correspond with the internalization of the Sytox Green, this can occur only with the permeability is altered, enhancing > 500-fold the fluorescence of the dye upon nucleic acid binding. Error bars represent the standard deviations (SD). Each data point indicates the mean of the results of three measurements. *Key*: C-, negative control (parasites without treatment); C+, positive control (parasites permeabilized with Triton X-100)

Cell death by necrosis is characterized by rupture of the plasma membrane. For this reason, plasma membrane integrity of the treated promastigotes was evaluated. Treatment with the different PHs did not alter the plasma membrane integrity of any *Leishmania* strain assayed by Sytox Green (Fig. 4), differently of Triton X-100 permeabilized promastigotes (positive control) of both strains, where an increase in fluorescence was detected from the beginning.

**Fig. 5:**
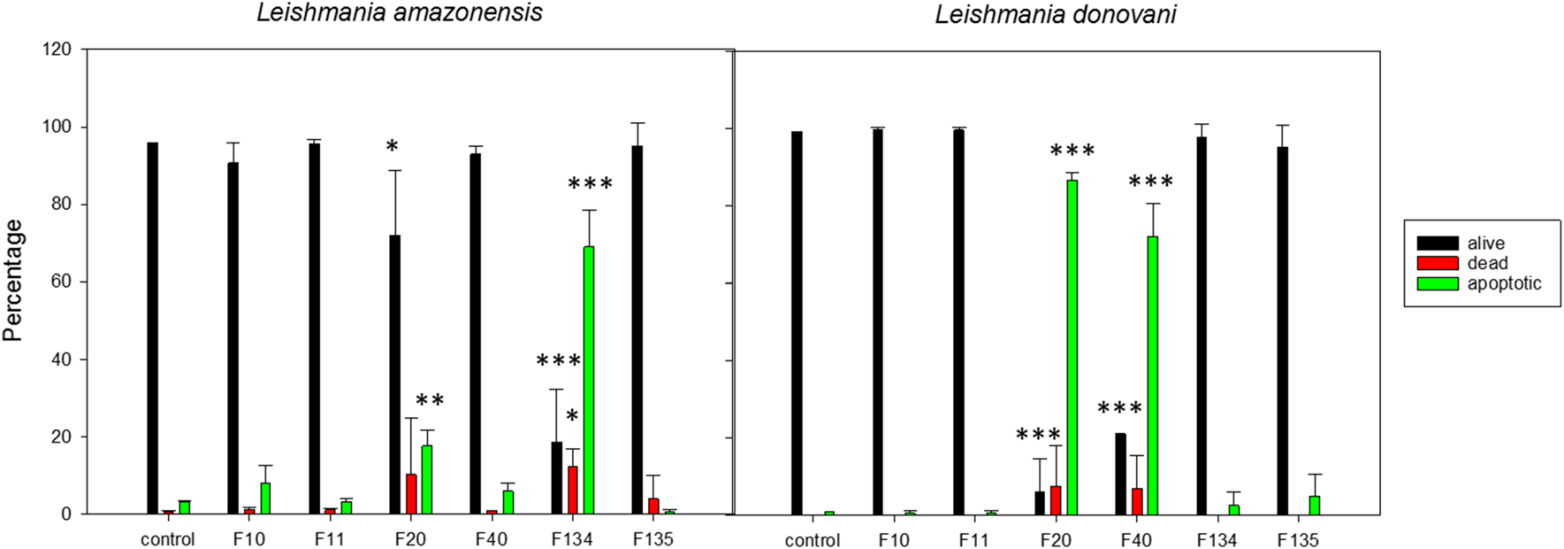
Phosphatidylserine exposure after 24 h of incubation with the IC_90_ of phenalenones. All data are given in percentages by the Tali^TM^ image-based cytometer (Invitrogen). Error bars represent the standard deviations (SD). Each data point indicates the mean of the results of three measurements. *P < 0.05, **P < 0.01, ***P < 0.001

On the other hand, we observed strong changes in the composition of the external part of the plasmatic membrane, with a higher amount of bounded annexin V after incubation of *L. amazonensis* with the phenalenone F134 (*F*_(6, 20)_ = 92.409, *P* < 0.001). In addition, when *L. donovani* was incubated with phenalenones F20 and F40 (*F*_(6, 20)_ = 248.643, *P* < 0.001 and *F*_(6, 20)_ = 248.643, *P* < 0.001, respectively), parasites also showed linked annexin V (Fig. 5). There were also statistically significant changes, not as strong in this case, in the bound annexin V when promastigotes of *L amazonensis* were incubated with the IC_90_ of F20 (*F*_(6, 20)_ = 92.409, *P* = 0.017). Despite this strong disturbance in the plasma membrane, the permeability of the parasites remained intact as observed, excluding cell death by necrosis. Together, these data indicate that the treatment with PHs induces distinct alterations associated with apoptosis-like cell death in *L. amazonensis* and *L. donovani* promastigotes.

### DNA alteration

**Fig. 6:**
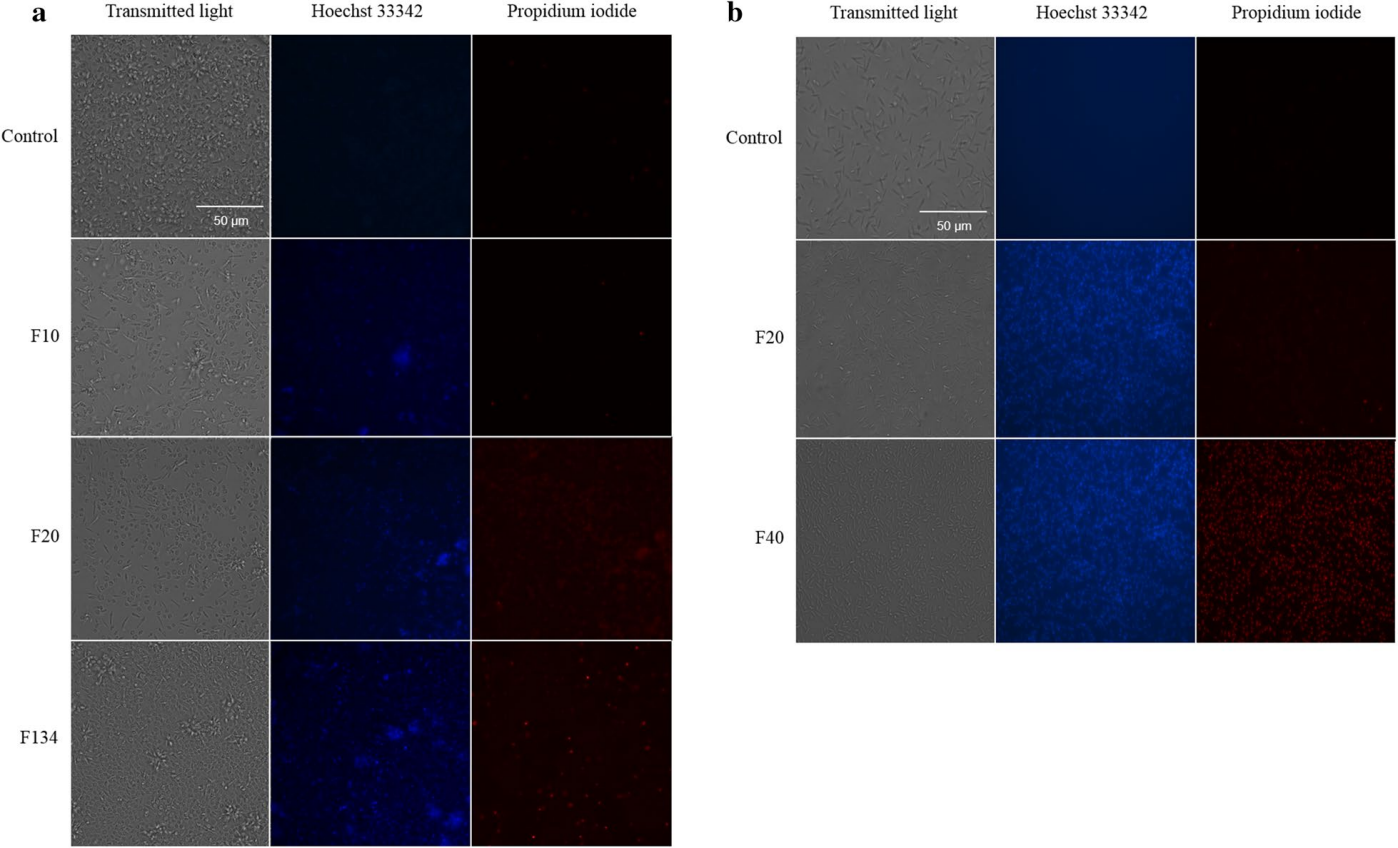
Hoechst-Propidium iodide staining. Results after 24 h of incubation with the IC_90_ of PHs. **a** *Leishmania amazonensis*. **b** *Leishmania donovani*. Images were captured using an EVOS FL Cell Imaging System (20×). *Scale-bar*: 50 µm

Finally, the double staining results were obtained after 24 h of incubation of the cells with the molecules at the IC_90_, and the two fluorescent dyes (Hoechst 33342/Propidium iodide). According to the properties of the dyes, cells stained with Hoechst 33342 were light blue when chromatin was in a normal state. Early apoptotic cells stained with Hoechst 33342 showed bright blue nuclei due to chromatin condensation. Non-viable cells stained with propidium iodide showed dense bright red nuclei. Figure 6 shows the images obtained when the assays were performed with *L. donovani*. It is clear that F20- and F40-treated parasites exhibited chromatin condensation (blue), at the same time that the negative control exhibited a soft uniform blue (Fig. 6). Meanwhile, *L. amazonensis* parasites showed a strong chromatin condensation (intense blue) when treated with the IC_90_ of the phenalenones F10, F20 and F134 (Fig. 6).

### Oxidative stress

**Fig. 7:**
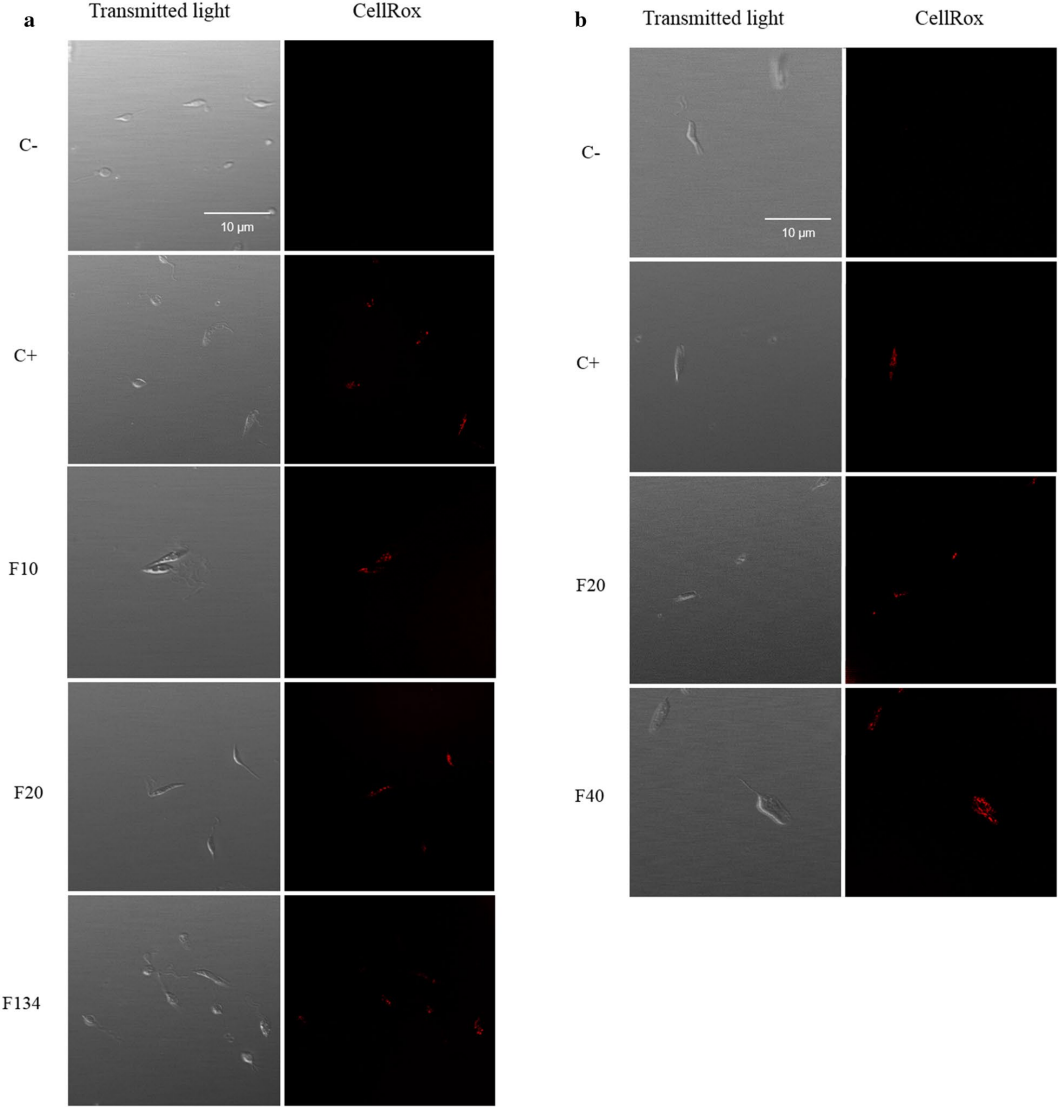
CellROX Deep Red staining. Results after 24 h of incubation with the IC_90_ of PHs. Red staining corresponds to ROS production. **a** *Leishmania amazonensis*. **b** *Leishmania donovani*. Images were captured using a Confocal Leica TSC SP (40×). *Scale-bar*: 10 µm

Visual fluorescence of CellROX Deep Red was used to assess the presence of reactive oxygen species (ROS) in promastigotes of *L. amazonensis* and *L. donovani* exposed to PHs. As expected, parasites without treatment (negative controls) did not display any increase in CellROX fluorescence compared to H_2_O_2_-treated positive controls. Treatment of *L. amazonensis* parasites with the IC_90_ of F10, F20 and F134 compounds for 24 h, increased the visual fluorescence of CellROX Deep Red compared to untreated cells when observed under the microscope (Fig. 7). Similar results were observed when *L. donovani* promastigotes were incubated with F20 and F40 PHs after 24 h of treatment.

## Discussion

Many PHs have been described recently to possess different biological activities ranging from antifungal to antiHIV. For example, Li et al. [28] described the antifungal activity of some PHs isolated from *Auxarthron pseudauxarthron* against *Cryptococcus* and *Candida* and Pang et al. [29] isolated some PHs with antiHIV activity from *Aspergillus*. Other studies have shown antibacterial activities against many species such as *Pseudomonas aeruginosa*, *Escherichia coli*, *Staphylococcus aureus* and *Bacillus subtilis* [30]. Finally, a number of studies have shown the ability of PHs to inhibit the growth of different protozoans such as *Leishmania amazonensis*, *L. donovani*, *Trypanosoma cruzi* [13, 18], as well as *Plasmodium* spp. and *Acanthamoeba* spp. [14, 15].

In addition, three other studies have gone deeper into the properties of the PHs as photosensitizer molecules and have studied the biological activity of the PHs together with light stimulation. Muehler et al. [16] compared the antibacterial activity of some PH with or without photodynamic therapy (PDT), consisting in a specific range of light spectra stimulation of the treated bacteria during 60 seconds; these authors not only could prove an increase in the activity of the PHs, but also an increase their cytotoxicity. Cieplik et al. [31] studied de PDT combined with PHs against biofilms and obtained activities similar to chlorhexidine; moreover, these authors analysed some of the events which occurred in the bacteria, observing damage in their plasmatic membranes. Their observations correspond with the data obtained in the present study, since we observe alterations of the plasma membrane permeability studied with the Sytox Green after treatment with the six compounds. Another recent study on PDT together with PH treatment carried out by Salmerón et al. [32] obtained antitumoral results in a human leukemia cell line. Furthermore, these authors studied the type of cell death induced by the combination PH-PDT, and the results indicated that the treated tumor cells suffered an apoptosis mechanism, demonstrated by DNA fragmentation, decrease in the mitochondrial membrane potential, chromatin condensation and ROS production among others. Although in the present study we did not use light stimulus during the treatment with PHs, the results of PDT showed a decrease in the IC_50s_, but probably an increase in the CC_50_s. Altogether, the results of Salmerón et al. [32] support the data shown in the present study, where we also suggest an apoptosis mechanism by a decrease in mitochondrial membrane potential, chromatin condensation, phosphatidylserine exposure and ROS production.

It has been demonstrated that good levels of ATP are necessary for the correct development of all the mechanisms involved in programmed cell death [33]. In a study of the possible effects of the compounds on the mitochondrial membrane potential, Freijo et al. [18] showed a decrease on the potential when *L. amazonensis* were incubated with phenalenone compounds (F10, F20, F40, F134 and F135), with the decrease being more pronounced with F10, F20 and F134. The results of the present study also showed a decrease in potential of *L. donovani* promastigotes when incubated with the IC_90_ of the phenalenones F20 and F40.

The special characteristics of these pathogenic protozoans is that they contain typical mitochondria as a single organelle, in comparison with mammalian cells possessing numerous, therefore, the proper function of the single mitochondrion in *Leishmania* is vital [34]. In addition, the fine structure of the mitochondrion may vary depending on the genus and species of parasite, and depending on environmental and nutritional resources available, the organelle can fill up to 12% of the cell volume [35].

In our study, the observed alteration levels of the mitochondrial potential in PHs-treated parasites did not reach significant low values when compared to the control thus indicating that the machinery of the mitochondria was still able to continue with its functions, which is in accordance with the maintained ATP levels mentioned above. Moreover, similar observations regarding the alteration levels of the mitochondrial potential, have been detected in previous studies even using the reference drug, miltefosine [36].

In live cells, phosphatidylserine is located on the cytoplasmic surface of the cell membrane, while in apoptotic cells it is translocated from the inner surface to the outer surface of the plasma membrane. Annexin V has a high affinity for this phospholipid, so parasites under apoptosis will be marked with annexin in the outer part of their plasma membrane, while the cytoplasm of dead or late apoptotic will be stained in red, and live cells will remain unstained. In addition to the results described in the membrane alterations section, it also seemed that compound F10 could induce the linkage to annexin V in the external membrane of *L. amazonensis* when incubated for more than 24 hours.

ROS induced by chemotherapeutic agents is closely associated with mitochondrial function, cytochrome *c* release, and apoptosis. Compounds such as PHs, could induce apoptosis through the mitochondrial pathway initiated by ROS production, therefore our proposal is that phenalenone structure compounds could induce the formation of ROS, and excessive ROS could trigger apoptosis by altering the mitochondrial membrane potential and damaging the respiratory chain, as it has been proved in a human leukemia cell line [32].

Previous research concerning the subject of ROS production and the consequent cell death, have reported that many of the current treatments for infections caused by kinetoplastids have the target or interfere with the metabolism of the trypanothione, the most common antioxidant of trypanosomatids and an analog of glutathione, which plays an important role in the elimination of free radicals in these organisms. For example, benznidazole as well as nifurtimox, the two current treatments for Chagas’ disease act in this step of the metabolism [37]. In addition, the first line treatment against African sleeping sickness, melarsoprol, causes the same interference with the trypanothione metabolism [38]. Moreover, pentavalent antimonials, the most common drugs used to treat leishmaniasis, have in their mode of action the capacity to inhibit the trypanothione reductase with the consequence of ROS accumulation, resulting in cell death [39].

Regarding the differential species-specific activity of PHs, based on the structure of the molecules, we can only suggest some hypotheses which require performing further experiments with similar structures. Our first suggestion is that the substituent *N*,*N*-dimethylethylenediamine is more selective against *L. amazonensis* and our second hypothesis is that *L. donovani* is more selective for substituents with a benzyl on the substituent.

Finally, concerning the fact that death mechanisms of trypanosomatids may be involved in pathogenesis, the identification of parasite-specific regulators could represent a rational and attractive alternative target for drug development for these neglected diseases [40]. To the best of our knowledge, our study shows for the first time that PHs could be promising molecules for future treatments against leishmaniasis.

## Conclusions

In this study, synthetic phenalenones F10, F20 and F134 induced several ultrastructural and morphological changes in promastigotes of *L. amazonensis* and synthetic phenalenones F20 and F40 induced similar changes in promastigotes of *L. donovani*. Mitochondrial membrane potential changes, ROS production, phosphatidylserine externalization, cell shrinkage, a rounded shape of the parasites, nuclear condensation and no change in membrane permeability were observed in parasites that were treated with the mentioned synthetic phenalenones. The major finding in the present study was that these PHs induced cell death in *Leishmania* spp., sharing several phenotypic characteristics with other cases of programmed cell death in metazoans. These findings contribute to elucidation of the alterations caused by phenalenones and development of new strategies for the study of their antileishmanial activity. However, further biochemical studies are needed to unveil the mechanism of action of these phenalenones in promastigotes of *L. amazonensis* and *L. donovani* in order to generate less toxic drugs for the treatment of leishmaniasis.

## Data Availability

All data generated or analysed during this study are included in this published article.

## Abbreviations

ABB: annexin binding buffer
DMSO: dimethyl sulfoxide
IC: inhibitory concentration
PH: 1H-phenalen-1-one
PCD: programmed cell death
PDT: photodynamic therapy
PI: propidium iodide
ROS: reactive oxygen species
WHO: World Health Organization
